# Using a basic life support stress-test to assess the influence of ethanol-based hand rubs nitril gloves and of physically demanding clinical tasks on personal protective equipment fit (GLOVDIS-1) – a pilot Manikin study

**DOI:** 10.3205/dgkh000666

**Published:** 2026-07-17

**Authors:** Tobias P. Taubner, Michael Bentele, Florian Salm, Dario Zocholl, Benjamin Taubner, Stefan Bushuven, Nico T. Mutters

**Affiliations:** 1 University of Bonn, University Hospital Bonn, Institute of Hygiene and Public Health, Bonn, Germany; 2 Training Center for Emergency Medicine (NOTIS e.V.), Pfronten, Germany; 3 Institute for Anesthesiology, Intensive Care, Emergency Medicine and Pain Therapy, Hegau-Bodensee Hospital, Singen, Germany; 4 Department of Interdisciplinary Emergency, Intensive Care Medicine and Anesthesia, St. Vinzenz Hospital, Pfronten, Germany; 5 Prevent Infect Akademie, Freiburg, Germany; 6 Institute for Medical Biometry, Informatics and Epidemiology, Medical Faculty, University of Bonn, Germany; 7 Institute for Psychology, University Bern, Switzerland; 8 Department of Anesthesiology and Critical Care, Medical Center, University of Freiburg, Germany; 9 Institute for Didactics and Educational Research in Medicine, Clinic of the University Munich, Ludwig-Maximilians-University, München, Germany

## Abstract

**Aim:**

Hand hygiene and personal protective equipment (PPE) are essential components for infection control. However, existing data are inconclusive regarding the impact of ethanol-based hand rubs on glove integrity, and evidence is limited on whether individual PPE components maintain adequate fit and usability during physically demanding clinical tasks. This pilot study aimed to evaluate the feasibility of a simulation-based protocol, designed to investigate hygiene- and PPE-related benefits and limitations under realistic emergency conditions.

**Methods:**

In a prospective observational study, 60 health care workers (HCW) pairwise performed two cycles of cardiopulmonary resuscitation (CPR) according to Basic Life Support standards while wearing full personal protective equipment. Participants were double-gloved; outer gloves were disinfected with an ethanol-based hand rub (EBHR), while the inner gloves remained native. Glove integrity was assessed according to DIN EN 455-1, and subjective PPE fit was rated before and after CPR. Feasibility, glove leakage, and changes in PPE fit were analyzed descriptively and exploratorily.

**Results:**

The study protocol proved feasible, with no dropouts and successful integrity testing of 478 out of 480 gloves. Glove leakage occurred in 3.8% of gloves, with no significant difference between disinfected and native gloves. Subjective PPE fit remained unchanged for gloves, respirators, and eye protective devices, whereas gown fit deteriorated significantly after CPR.

**Conclusion:**

This pilot study demonstrates the feasibility of a standardized simulation-based Basic Life Support stress test to investigate the influence of glove disinfection with two different EBHRs on PPE performance under simulated emergency conditions. Ethanol-based glove disinfection did not increase glove leakage, whereas gown fit deteriorated during CPR. Larger, adequately powered studies may base on these results to further evaluate PPE performance and inform evidence-based infection prevention strategies in emergency care.

## Zusammenfassung

**Zielsetzung:**

Händehygiene und persönliche Schutzausrüstung (PSA) sind zentrale Pfeiler der Infektionsprävention. Ob und inwieweit Ethanol basierte Händedesinfektionsmittel medizinische Handschuhe im klinischen Alltag beeinträchtigen ist unklar, die Datenlage ist gemischt. Zudem fehlen Studien, ob einzelne PSA-Komponenten bei körperlich belastenden klinischen Tätigkeiten ihre Passform und Funktionalität beibehalten und in welchem Umfang sie die Arbeit des Nutzers behindern. Ziel dieser Pilotstudie war die Prüfung der Durchführbarkeit eines simulationsbasierten Protokolls zur zukünftigen Evaluierung hygienerelevanter und PSA-bezogener Aspekte unter realitätsnahen Notfallbedingungen.

**Methode:**

In einer prospektiven Beobachtungsstudie führten 60 Mitarbeitende des Gesundheitswesens paarweise zwei Zyklen kardiopulmonaler Reanimation (KPR) gemäß Basic-Life-Support-Standard unter vollständiger PSA durch. Die Teilnehmenden trugen je zwei Paare Handschuhe übereinander; die äußeren Handschuhe wurden mit einem Ethanol basierten Händedesinfektionsmittel desinfiziert; die inneren Handschuhe blieben unbehandelt. Anschließend wurde die Integrität der Handschuhe gemäß DIN EN 455-1 geprüft. Wie subjektiv funktional die PSA sitzt, wurde vor und nach der KPR erfasst. Durchführbarkeit, Defekte in den Handschuhen sowie Änderungen im Sitz der PSA wurden deskriptiv und explorativ ausgewertet.

**Ergebnisse:**

Das Studienprotokoll erwies sich als durchführbar; es traten keine Dropouts auf, bei 478 von 480 Handschuhen konnte eine Integritätsprüfung erfolgen. Leckagen wurden bei 3,8% der Handschuhe ohne signifikanten Unterschied zwischen desinfizierten und unbehandelten Handschuhen festgestellt. Der subjektiv empfundene Sitz blieb für Handschuhe, Atemschutzmasken und Augenschutz unverändert, während sich die Passform der Schutzkittel nach der CPR signifikant verschlechterte.

**Schlussfolgerung:**

Die Studie zeigt die Durchführbarkeit des standardisierten, simulationsbasierten Basic-Life-Support-Stresstests zur Evaluation der Fragestellung unter simulierten Bedingungen. Die Handschuhdesinfektion führte nicht zur erhöhten Defektrate von Nitrilhandschuhen, während sich die Kittelpassform während der KPR verschlechterte. Größere, adäquat geplante Studien sind erforderlich, um den Nutzen einzelner PSA-Komponenten weiter zu erörtern und evidenzbasierte Empfehlungen für die Infektionsprävention in der Notfallversorgung zu generieren.

## Introduction

Hand antisepsis is a cornerstone of patient safety [1], yet its consistent implementation in clinical practice remains challenging [2]. This challenge is particularly pronounced in emergency settings, where hand antisepsis is often suboptimal [3], despite vulnerable patients being at increased risk for hospital-acquired infections [4]. These situations are frequent: In Germany alone, emergency medical services recorded 7,804,180 deployments and emergency department treated over 12,000,000 patients in 2023 [5], [6].

Although not all emergencies are peracute or life-threatening, a substantial proportion are. Annual Germany statistics report high numbers of cardiovascular emergencies such as stroke (260.000) [7], myocardial infarction(186,100) [8], sepsis (91,000) [9] and trauma-related injuries (approximately 10 million) [10] – all of which place patients at increased risk for health care acquired infections (HAIs).

These infections worsen patient outcomes, undermine the efforts of high-performance teams, and generate considerable economic and ecological burdens through inefficient resource use. While hand antisepsis cannot prevent all HAIs, it has shown to reduce their incidence [11].

Infection prevention and control are essential not only for patient safety but also for protecting health care workers (HCW) in both clinical and preclinical settings. However, in emergency situations, infection control is necessarily secondary to immediate life-saving interventions. For HCW who frequently manage emergencies, maintaining core skills such as cardiopulmonary resuscitation (CPR) and basic life support (BLS) under stress is paramount. Even for experienced teams, infection control measures must therefore be feasible without impairing CPR performance.

One potential strategy to include hand antisepsis into emergencies is glove disinfection [12], [13], [14]. While an increasing number of studies suggest that alcohol-based hand rubs (ABHR) may be used to disinfect gloves [14], [15], [16], this practice remains controversial. The Commission for Infection Prevention and Hygiene in Healthcare and Nursing (KRINKO) [12] recommends that glove disinfection should only be considered in exceptional situations when changing gloves would significantly impair workflow, and only if the gloves are certified as chemically resistant (EN 374). Moreover, gloves must be replaced immediately in cases of visible perforation, contamination with blood, secretions or excretions, exposure to non-enveloped viruses, and after use during patient washing procedures. At the same time, glove durability represents a critical safety factor for HCW. Several studies suggest that disinfected nitrile gloves may exhibit increased perforation rates [16], reduced tensile strength [17], and faster tearing [18], potentially compromising the protective barrier for both staff and patients. 

CPR offers a pragmatic method to assess mechanical stress on gloves in clinical contexts. Cardiac arrests frequently occur in intensive care units, operating rooms, and emergency departments [19], and CPR follows a highly standardized algorithm [20]. When manual chest compressions are performed, substantial mechanical load is applied to the hands, which may increase the risk of glove perforations. 

Since the COVID-19 pandemic, the interaction between CPR, hand antisepsis, and personnel protective equipment (PPE) has gained renewed relevance. HCW increasingly perform resuscitation while wearing extensive PPE – double gloves, gowns, respirators and eye protective devices – particularly when caring for patients with suspected or confirmed COVID patients [21], accompanied by intensified disinfection efforts [22].

Beyondbarrier integrity, the fit and comfort of PPE represent additional changes. A 2020 Cochrane review by Houghton et. al. [23] reported that physical discomfort reduces adherence to infection control guidelines and that many HCW experience PPE-related symptoms such as fatigue, breathing difficulties, and excessive sweating. However, evidence on how PPE fit and usability change during or after physically demanding tasks such as CPR remains understudied. Yet empirical it can be assumed that the comfort and fit of PPE deteriorate over time.

Performing effective, patient- and personnel-safe CPR while maintaining infection control under realistic conditions remains challenging. To address this, we developed a simulation-based study protocol designed to assess glove integrity after ethanol-based disinfection and changes in PPE fit during CPR. This pilot study aimed to test the feasibility of this protocol and to generate preliminary data for hypothesis development, effect size estimation, and the planning of future adequately powered studies.

The following hypothesis should be tested:

- Main finding: The pilot test protocol is feasible regarding dropout rate, testing gloves due to DIN EN 455-1 [24], and feedback from participants and the CPR instructor,
- Exploratory analysis I: Disinfection of nitrile gloves by EBHR do not hold as tight as native gloves after CPR tested by DIN EN 455-1 [24],
- Exploratory analysis II: The PPE (double gloved, gown, N95 respirator, eye protective devices) fits worse after cardiopulmonary resuscitation tested by the users subjective evaluation.

## Methods

### Study design

We conducted a prospective observational study of HCW as pairs performing cardiopulmonary resuscitation in full protection (double gloved, FFP2 respirator N95-equivalent, according to EN 149:2001+A1:2009, gown, eye protective devices) on a resuscitation manikin. Double gloving means, that a participant wears two gloves on one hand, an inner one and an outer one.

### Setting

We used rooms at the hospitals in Constance and Singen, the German Red Cross rescue stations in Singen, Radolfzell and Constance, as well as private rooms as test locations. 

The study included HCW from both hospital and prehospital settings, familiar with basic life support.

### Recruitment

Recruitment and testing took place from October 2022 to July 2023.

Participants were recruited via email invitations sent to full-time staff at the GLKN hospital and local emergency medical services (red cross district Konstanz, Malteser Konstanz), as well as through private outreach. Eligible participants were fully qualified or in the final year of their training.

The sample included emergency medical technicians paramedics (EMT-P), EMT basics, EMR/First Responder, certified anesthesia assistants, registered nurses, medical students and physicians.

Exclusion criteria were age under 18 and refusal to participate.

### Implementation

Participants were tested in pairs (dyads), based on availability rather than predefined characteristics. 

During the study an observer was present and assessed the participants using paper-based checklists. One checklist comprised core quality objectives for cardiopulmonary resuscitation, adapted from the American Heart Association. Additionally, we assessed donning and doffing of PPE.

### Materials

The PPE consisted of double nitrile (CAS 9003-18-3) gloves of 0.05–0.14 mm from different manufacturers (see Table 1 (Tab. 1)), FFP2 respiratory masks (GVS S.p.A., Bologna, Italy), gowns (Medi-Inn Online GmbH, Hirten, Germany; Medline International Switzerland Sàrl, Geneve, Switzerland), eye protective devices (Adolf Wuerth GmbH & Co.KG, Künzelsau-Gaisbach, Germany). As a resuscitation manikin Laerdal Anne (Laerdal Medical AS, Stavanger, Norway) was used and as EBHR Aseptoman MED (ethanol 65%, Dr. Schumacher GmbH, Malsfeld, Germany) or Aseptoman Forte (ethanol 85%, Dr. Schumacher GmbH, Malsfeld, Germany). 

Dyads performed two cycles of cardiopulmonary resuscitation according to BLS on the manikin while wearing full protection. The observer assessed both cycles according to our AHA-based cardiopulmonary resuscitation checklist, consisting of 13 equally weighted items. 

Due to the (simulated) anticipated high infectious risk setting the participants were told not to do any kind of ventilation, like mouth-to-mouth ventilation.

First the participants got instructions on data protection and withdrawal rights.

Afterwards the participants started with donning PPE, while being assessed by the observer with the checklist.

After donning PPE all participants disinfected their outer gloves with an EBHR.

In the following out-of-hospital emergency scene, the first person to arrive was sent to the manikin for checking the manikin's responsiveness, breathing and pulse, as well as for initiating the rescue chain.

Subsequently, this person started chest compressions without creating a no-flow time. After two to three cycles of compression (one cycle 30 compressions), the second person was sent into the situation, equipped with an automated external defibrillator (AED). As an out-of-hospital setting with limited resources, a bag mask device was not available at this point.

The second person was ordered to pass the AED to the first responder and take over chest compressions. Following this the first person switched on the AED, applied the AED electrodes correctly, performed the heart rhythm analysis with the AED, ordered the other participant to step back before the shock and performed the shock safely. Thereafter, the immediate resumption of compressions was expected.

**Figure 1:**
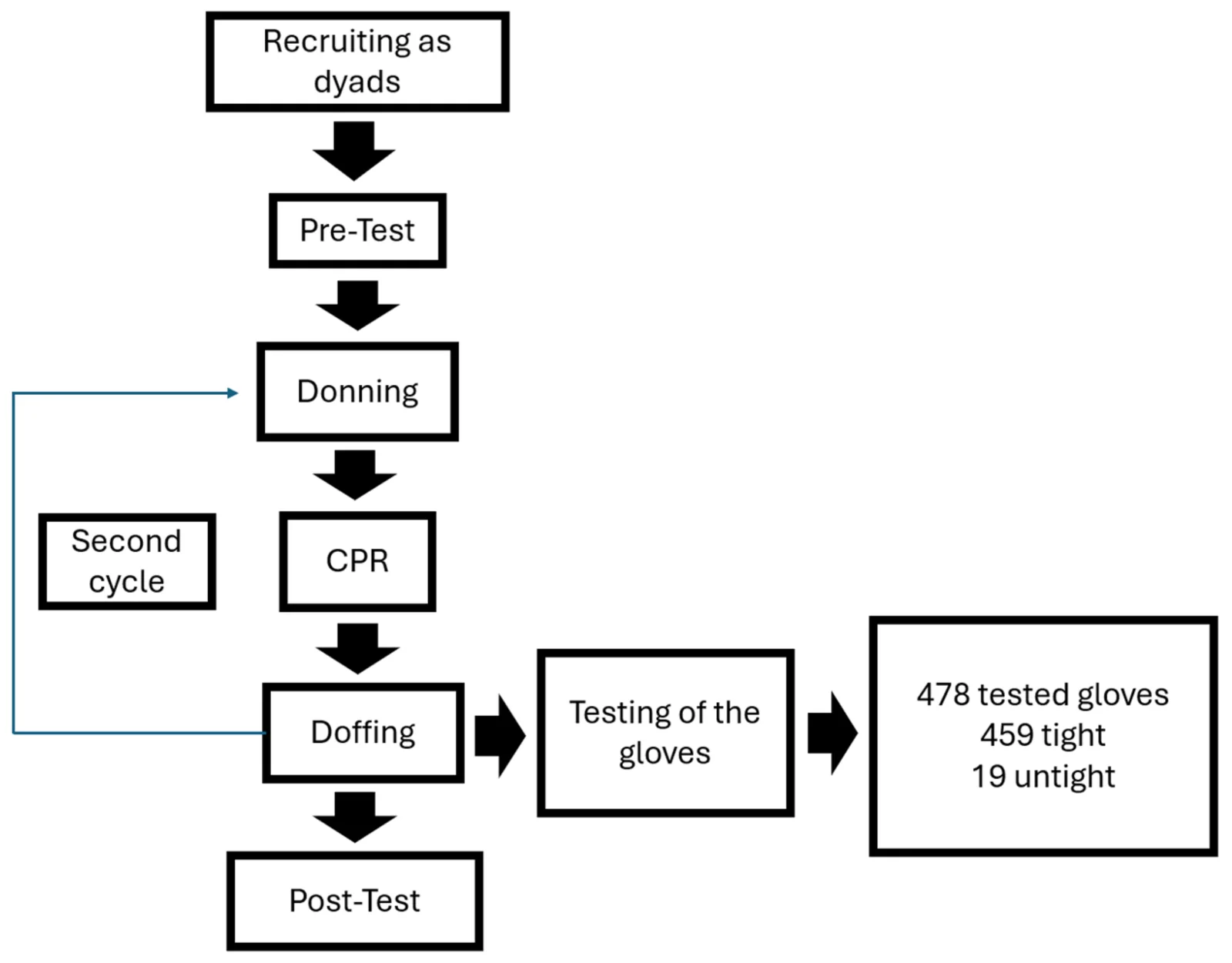
Schema of the pilot study protocol.

BLS was followed by doffing PPE. Once more the participants got assessed by the observer according to the checklist. By donning again, the second cycle began (Figure 1 (Fig. 1)).

If the PPE stayed undamaged in round one, mask, gown and eye protective devices got used again in round two. The participants got two new pairs of gloves. The gloves of round one got collected for subsequent analysis. In the second cycle of cardiopulmonary resuscitation, the participants swapped roles as first and second responders.

We used labeled trays to identify the gloves. Each tested glove was assigned to a specific participant (anonymous numbers), to either the left or right hand, and to whether it was worn as an inner native or outer disinfected glove. The main investigator tested all gloves for leaks in accordance with DIN EN 455-1 [24]. Therefore, we used an apparatus with six integrated Plexiglas tubes. The gloves got pulled over the tubes. Complying to DIN-EN 455-1 [24] we filled each glove with 1,000 ml water, followed by surveying for 10 minutes. Non-tight gloves were identified by water leakage.

In the second cycle of CPR, the participants swapped roles as first and second responders.

In addition, the participants rated the fit of their gloves, protective gown, respirator masks and eye protective devices on an analog scale of 1–10 (1=poorest fit, 10=best fit) before and after performing CPR.

The rating was based on wearing comfort and the participant’s subjective feeling of protection provided by the PPE.

### Statistics

Data analysis was conducted with R and RStudio, version 2024.12.1+563. Therefore, we used pwr, psych, exact2x2, summarytools, skimr, ggplot2, effectsize, rstatix, coin, dplyr and tidyr as packages. Results were validated with SPSS, version 29.0.1 IBM (IBM, Armonk, NY, USA). 

Feasibility was not tested statistically, but assumed in case of preterm quitting the scenario, setting problems, negative feedback by participants and problems in conducting the DIN tests.

Concerning the secondary hypotheses, exploratory analysis of glove tightness was conducted using an exact McNemar test comparing tightness (yes/no) of inner and outer gloves. For pre-post comparisons of subjective protection by PPE Wilcoxon signed-rank test with estimation of the rank biserial coefficient was used. The significance level was set to a=0.05. Given the exploratory nature of the study, no adjustment for multiplicity was applied.

### Ethical approval

The ethics committee of the Baden-Württemberg Medical Association approved the study (05.10.2022, registration number: F-2022-101).

## Results

### Participants

60 HCWs (35 male, 25 female) took part, forming 30 pairs. 

30 worked full-time in emergency medical services (EMS), 12 in nursing and 9 were physicians or 3^rd^ to 7^th^ year med students. A further 8 participants worked part time or voluntary in EMS. One participant was medical safety expert.

No participant dropped out during or after data collection.

### Main finding

The pilot study protocol proved feasible, functioning as a practical skill stress-test to assess disinfected gloves with EBHRs and evaluate PPE fit after performing CPR.

Data collection proceeded smoothly. Each trial lasted approximately 60 minutes for participant testing (document completion and CPR) and additional 45 min for glove testing according to DIN455-1 [25].

Collecting the gloves after doffing and correctly assigning them to the respective participant and hand during DIN EN 455-1 [25] testing was straightforward, thanks to labeled containers. During the physical tests (donning, CPR, doffing) no glove tore. Only two gloves ripped when being pulled over the plexiglass tube und were therefore excluded from the statistical analysis.

Of 480 collected gloves, we tested 478 (99.6%) successfully. Two gloves (0.4%) were artificially lost during the leakage test, tearing while applied to the testing device.

Participants reported no difficulties with the study protocol, nor did they complain about physical or psychological impairment. No participants preterm cancelled participation. 

Consequently, we approved hypothesis 1. However, following issues were obtained, that may be relevant for subsequent study protocols.

Feedback included the use of eye protective devices in individuals wearing prescribed glasses. Three people mentioned being distracted by glasses under their eye protective devices.

Recruitment also posed challenges, as most potential participants were employed full-time. 

Only a few responded to email invitations, while the majority were recruited through personal contacts.

### Glove integrity

Of 480 gloves, 2 were excluded as they were damaged during fitting to the apparatus (see above), 19 gloves (4%) were damaged; 9 (1.9%) outer gloves (disinfected) and 10 (2.1%) inner gloves (native). 

Regarding glove combinations rather than individual glove, we found that in 219 cases (92%), both the inner and outer gloves were leak-proof. In 9 cases (3.8%), only the outer glove was damaged while the inner remained intact. Conversely, in 10 cases (4.2%) the inner glove was damaged while the outer was not. In no instance were both the inner and outer glove simultaneously damaged.

Summing up round 1 we found lefthanded five cases with one glove damaged and one intact (3%), while on the right side only four cases of this constellation were found (2.4%).

In round 2 we found 4 cases (2.4%) on the left side with one damaged inner or outer glove (Figure 4) and six cases (3,6%) on the right side.

Exact McNemar tests showed no statistically significant differences between inner and outer glove integrity in any comparison (round 1 left: p=1.000; round 1 right: p=0.625; round 2 right: p=0.6875) while the comparison for round 2 left could not be estimated (Table 2 (Tab. 2)). 

Thus, the null hypothesis could not be rejected and therefore no sufficient evidence was found to support hypothesis 2.

### PPE fit

In round 1, participants rated the protective fit and comfort of gloves (pre mean: 8.13, post mean: 8.17), N95 respirators (pre mean: 7.47, post mean: 7.63) and eye protective devices (pre mean: 6.96, post mean: 6.54). Paired Wilcoxon signed-rank tests showed no statistically significant changes for gloves (p=0.988), N95 respirators (p=0.964) or eye protective devices (p=0.317).

In contrast, gowns were rated higher before CPR (pre mean: 7.67) than after (post mean: 7.17), with the Wilcoxon test indicating a statistically significant difference (p=0.002) (Table 3 (Tab. 3)).

In round 2, no statistically significant changes were observed for gloves (pre mean: 8.05, post mean: 7.90, p=0.159) or N95 respirators (pre mean: 7.78, post mean: 7.63, p=0.152), consistent with findings from round 1 (Table 4 (Tab. 4)).

In contrast, ratings for protective gowns decreased significantly after CPR (pre mean: 7.60, post mean: 6.77, p<0.001). A smaller but statistically significant decrease was also found for eye protective devices (pre mean: 6.74, post mean: 6.43, p=0.014), which was not observed in round 1 (Table 4 (Tab. 4)).

Overall, hypothesis 3 was confirmed for protective gowns and, in round 2, additionally for eye protective devices.

## Discussion

The feasibility of the study protocol and generated data for planning and conducting further studies on infection control and prevention was demonstrated. Within the pilot setting, no differences between tightness of disinfected and native gloves after basic life support (BLS) was detected, nonetheless impairment of PPE fit following BLS was observed. Our findings contribute to future research in several ways.

While laboratory studies have investigated the effects of EBHR on gloves, and others have addressed PPE effectiveness and comfort, standardized and reproducible procedures to assess these factors under clinical conditions remain scarce. This gap is particularly relevant for emergency and critical care settings, especially during infectious outbreaks and pandemics.

Our protocol provides a systematic framework for future studies to investigate benefits, limitations, and weaknesses of PPE and hand hygiene practices under realistic conditions.

Notably, no simultaneous perforation of both inner and outer gloves on the same hand occurred in the same round. While double gloving reduces clinical risk, defects become more consequential in routine single-glove use. As no statistically significant difference was detected, any true effect of disinfection on glove integrity is likely small. Consequently, a type II error cannot be excluded, underscoring the need for larger trials.

Importantly, our findings allow estimation of sample sizes required for adequately powered follow-up studies. Based on the observed leakage rate (3.8%), our sample size (n=480 gloves) would only allow detection of large effects (odds ratio ≥6.5; α α=0.05, power=0.9). Detecting more clinically relevant differences (e.g., OR=2.0) would require substantially larger sample sizes, potentially in the range of several thousand gloves. The exact number depends on the planned study design, desired power, alpha level, allocation ratio and the tests used.

Evidence on glove integrity after disinfection is mixed. Laboratory studies showed reduced tear resistance [18], decreased tensile strength and altered elasticity [17], and increased perforation rates [16] following alcohol-based disinfection. In contrast, other studies using standardized leak tests such as DIN EN 455-1 [16] or ASTM D5151-19 [25] found no perforations after ethanol-based disinfection.

Overall, our data suggests that glove rupture is a rare event for both disinfected and native gloves. This risk must be considered in the broader context with HAIs, which are rare per individual contact but cumulatively relevant given the large number of patient encounters and their associated morbidity and mortality. For instance, a 4% leak rate would extrapolate to 20,000–40,000 damaged gloves annually in a large hospital using up to one million gloves per year [26]. Although not every defect leads to transmission and not every transmission to an infection, low event probabilities may still become relevant when multiplied across large numbers of patient contacts. Additionally, in high consequence infectious diseases (HCID) like Ebola or Marburg-disease, tolerance for PPE failure is particularly low.

Subjective fit ratings for gloves, N95 respirators and eye protective devices did not change during BLS, whereas gown fit deteriorated statistical significantly. A safe and comfortable fit of personal protective equipment (PPE) is essential to protect HCW from infectious diseases as well as from chemical hazards.

Houghton et al. [23] emphasize that properly fitting, high-quality PPE is essential for preventing contamination. As most PPE is designed around average white male body proportions [27], frequent reports of discomfort [23] and perceptions of poor fit and insufficient protection among HCW are unsurprising [28]. 

Evidence on PPE fit and protective performance during and after healthcare tasks remains limited. A 2020 Cochrane review of 24 studies found that although increased body coverage may improve protection, evidence regarding the specific contribution of individual PPE components is of very low certainty. Furthermore, the use of additional PPE is associated with reduced comfort and greater difficulty during donning and doffing [29]. These limitations are not purely subjective: PPE can impose physiological burdens such as increased body and skin temperature and elevated heart rate [30], [31], and may also negatively affect patients by contributing to feelings of isolation.

Nevertheless, high-quality PPE and appropriate training remain essential for infection prevention and control [23]. In our study, participants rated gowns more negatively after CPR in both rounds, and eye protective devices were rated lower in the second round, suggesting that fit and comfort may deteriorate during physically demanding tasks. While PPE remains a cornerstone of infection prevention, further data are needed on the effectiveness of individual items, alongside improved designs that balance comfort and protection. 

## Limitations

Simulation-based studies cannot fully replicate real-world conditions; however, they provide standardized and reproducible settings while reducing the risk of adverse events [32], [33]. We focused on BLS, which involves comparatively low hygiene demands. Advanced life support procedures such as intravenous access, drug administration, or airway management may pose different risks and should be addressed in future studies. BLS was deliberately chosen as a prototypical emergency scenario because it is physically demanding, imposes substantial mechanical stress on gloves, and is frequently performed in clinical practice.

Regarding PPE, gloves from seven manufacturers were used without stratification by brand, size, or product line. While the two disinfectants used differed in ethanol concentration, both were ethanol-based formulations without additional alcohols such as propanols or other additives. This heterogeneity limits comparability but reflects real-world crisis conditions with variable supply chains. All gloves were nitrile and EU-certified (CE) with broadly comparable material properties. In addition, only one brand of each PPE item (except gloves) was tested. Although multiple glove brands were included, brand-specific performance and user ratings were not evaluated. 

As acceptable quality levels (AQL) vary between manufacturers, we did not assess AQL in this feasibility-focused pilot study. Accordingly, pre-existing glove defects cannot be excluded, as production standards permit a defined proportion of defective gloves. Future studies should therefore include batch-quality assessments.

The small sample size limits statistical power and generalizability. With our data (n=480 gloves, α=0.05, power=0.9), only large effects (odds ratio ≥6.5) could have been detected. The study was designed as a feasibility pilot to generate effect-size estimates rather than to test non-inferiority or equivalence.

Potential confounders such as sex, hand size, body weight, or use of bag-mask ventilation were not analyzed and should be considered in future studies. Recruitment was more challenging than anticipated, integrating the protocol into routine training or offering incentives could improve participation but may introduce selection bias. Participants were volunteers and may therefore have been more motivated, experienced, or receptive to new practices, while less motivated staff may have been underrepresented. In addition, awareness of being observed may have led to more cautious behavior (Hawthorne effect).

Some participants wearing prescription glasses reported difficulties with eye protection, highlighting the need for testing multiple models in future studies. PPE wearing time was relatively short (10–20 minutes), reflecting routine clinical practice but not prolonged use in certain situations. Furthermore, undamaged PPE items were reused in the second round due to logistical constraints, which does not reflect standard practice. 

Finally, subjective comfort ratings were not validated against objective assessments. Together, these limitations restrict the generalizability of our findings and underline the need for larger, standardized studies with defined glove-disinfectant combinations.

## Conclusion

In this pilot study, the study protocol for a simulated BLS stress test comparing gloves with or without disinfection with EBHR was tested successfully. No difference between native and disinfected gloves was observed, with a need for larger sample sizes in subsequent trials. Further, the study demonstrated that certain items of PPE may not maintain proper fit during BLS, bearing possible implications for HCW, manufacturers, BLS instructors, and trainers preparing HCW for work with hazardous materials.

Moreover, identifying which PPE components are essential versus those that may impede clinical tasks without providing additional protection remains important.

As epidemics, pandemics, and other transmissible hazards accompany the future of humanity, continued efforts to refine preparedness and to build robust evidence on PPE performance under demanding conditions remain inevitable.

## Notes

### Authors’ ORCIDs

- Taubner TP: https://orcid.org/0009-0006-5664-3701
- Bentele M: https://orcid.org/0000-0001-8592-6352
- Salm F: https://orcid.org/0009-0002-7607-1350
- Zocholl D: https://orcid.org/0000-0002-9218-6919
- Bushuven S: https://orcid.org/0000-0001-6272-0714
- Mutters NT: https://orcid.org/0000-0002-0156-9595

### Authors’ contribution

Stefan Bushuven and Nico T. Mutters contributed equally.

### Ethical approval

The ethics committee of the Baden-Württemberg Medical Association approved the study. 

### Funding

This study received no external funding.

### Competing interests

The authors declare that they have no competing interests.

## Figures and Tables

**Table 1:**
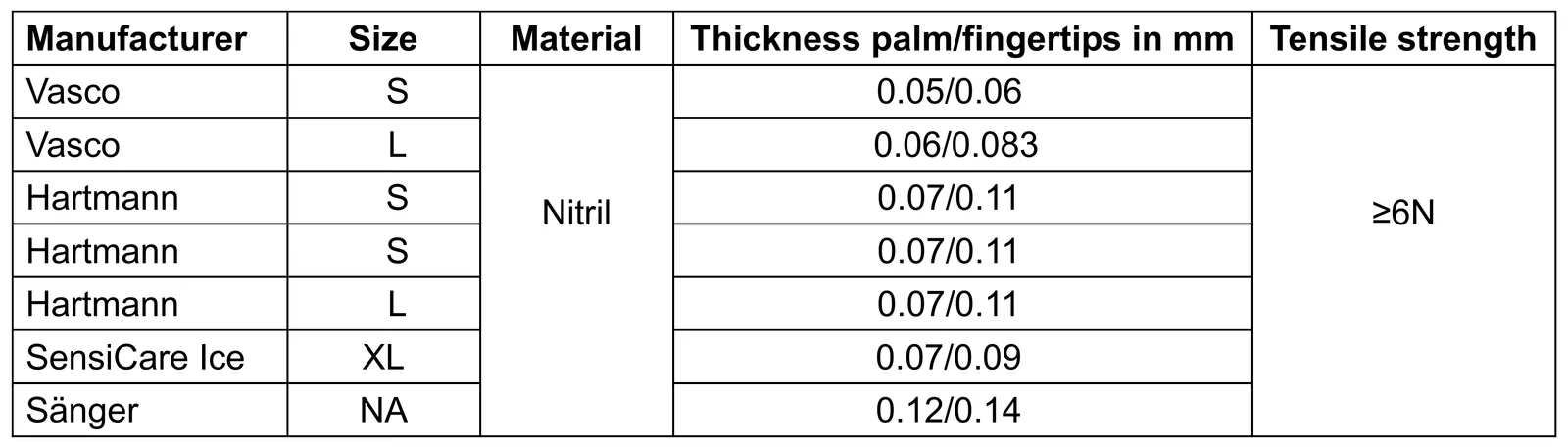
Manufacturer of used gloves

**Table 2:**
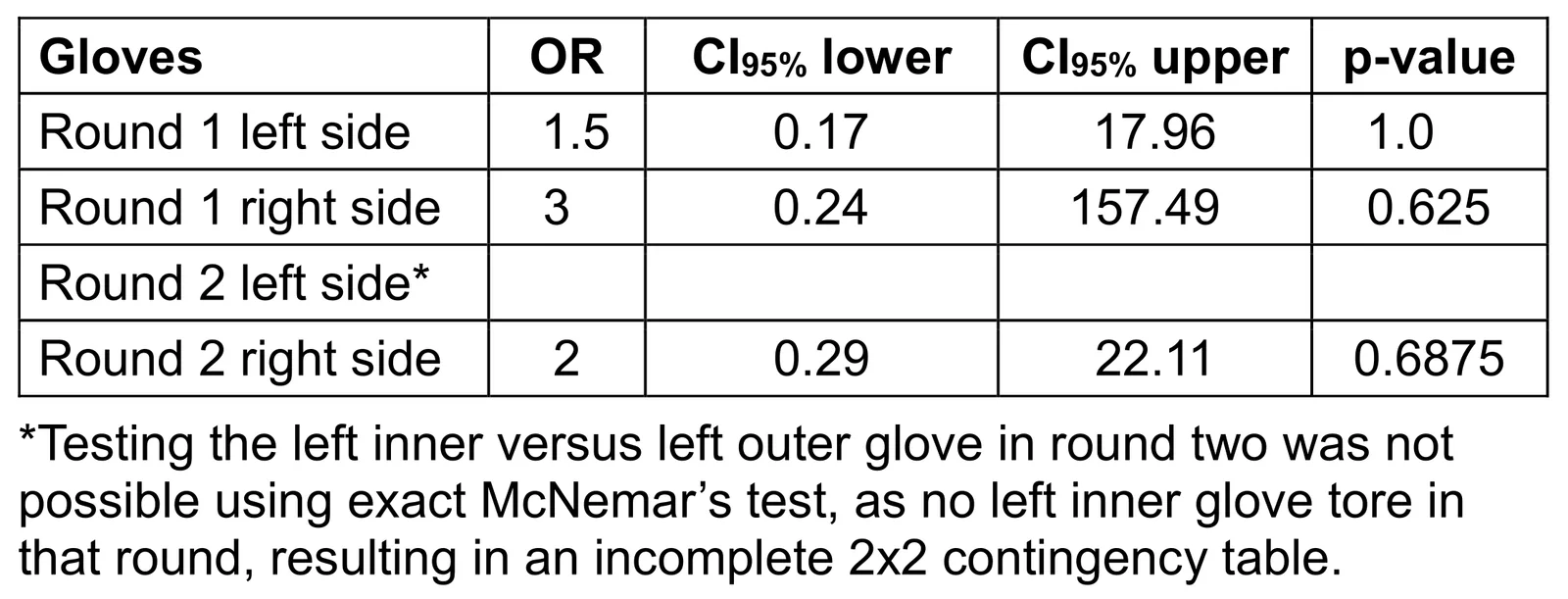
Tightness of gloves (Odds-ratios (OR), lower and upper 95% confidence intervals (CI), and p-value from exact McNemar tests)

**Table 3:**
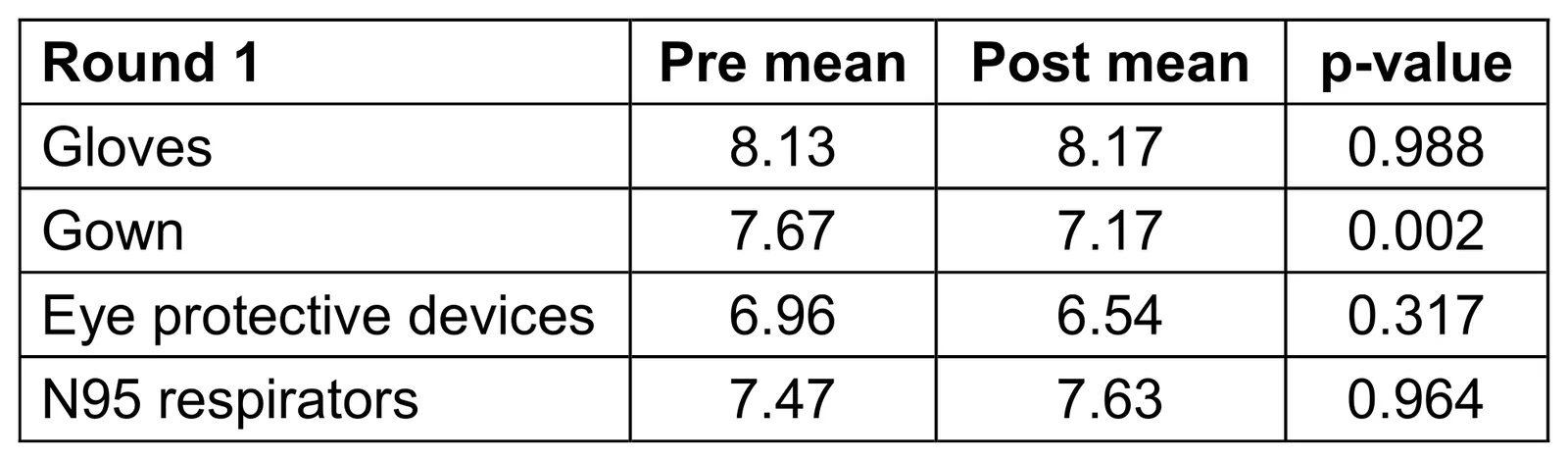
Means and p-values of ratings of PPE-fit round 1 (Wilcoxon, RStudio)

**Table 4:**
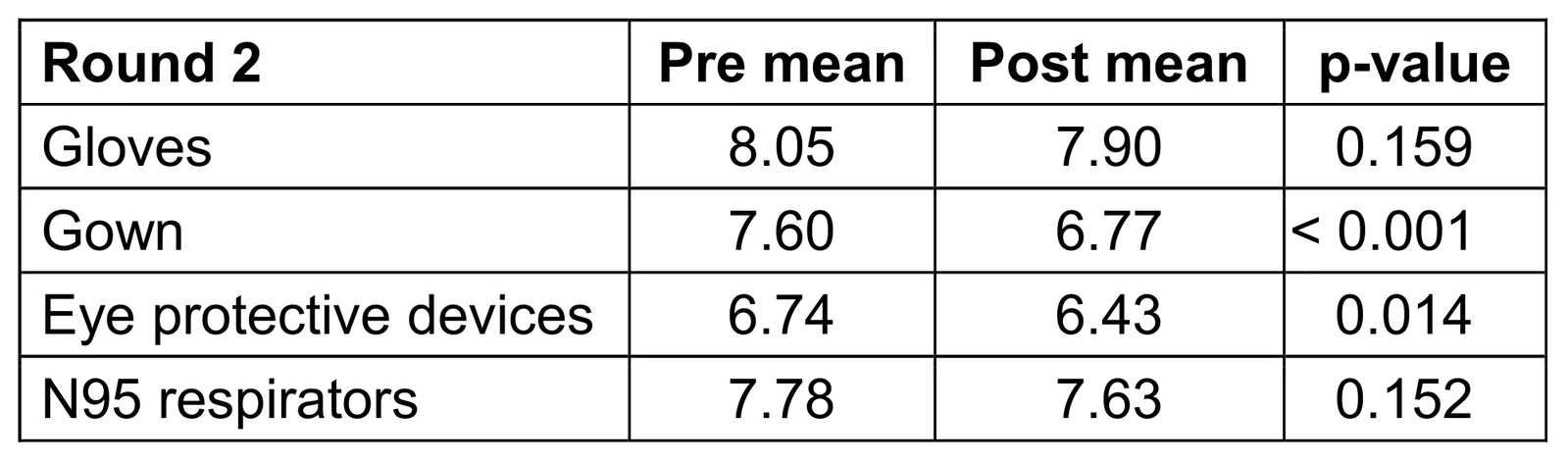
Means and p-values of ratings of PPE-fit round 2 (Wilcoxon, RStudio)
